# Expression Profile of *Glossina pallidipes* MicroRNAs During Symptomatic and Asymptomatic Infection With *Glossina pallidipes* Salivary Gland Hypertrophy Virus (Hytrosavirus)

**DOI:** 10.3389/fmicb.2018.02037

**Published:** 2018-09-03

**Authors:** Irene K. Meki, İkbal A. İnce, Henry M. Kariithi, Drion G. Boucias, Orhan Ozcan, Andrew G. Parker, Just M. Vlak, Monique M. van Oers, Adly M. M. Abd-Alla

**Affiliations:** ^1^Insect Pest Control Laboratory, Joint FAO/IAEA Programme of Nuclear Techniques in Food and Agriculture, International Atomic Energy Agency, Vienna, Austria; ^2^Laboratory of Virology, Wageningen University and Research, Wageningen, Netherlands; ^3^Department of Medical Microbiology, School of Medicine, Acıbadem Mehmet Ali Aydınlar University, Istanbul, Turkey; ^4^Department of Biostatistics and Medical Informatics, Acıbadem Mehmet Ali Aydınlar University, Istanbul, Turkey; ^5^Biotechnology Research Institute, Kenya Agricultural and Livestock Research Organization, Nairobi, Kenya; ^6^Entomology and Nematology Department, University of Florida, Gainesville, FL, United States

**Keywords:** Hytrosaviridae, *Glossina*, miRNA, immune system, GpSGHV, sterile insect technique, symptomatic, asymptomatic infection

## Abstract

The *Glossina pallidipes* salivary gland hypertrophy virus (GpSGHV) infects tsetse flies predominantly asymptomatically and occasionally symptomatically. Symptomatic infections are characterized by overt salivary gland hypertrophy (SGH) in mass reared tsetse flies, which causes reproductive dysfunctions and colony collapse, thus hindering tsetse control via sterile insect technique (SIT). Asymptomatic infections have no apparent cost to the fly’s fitness. Here, small RNAs were sequenced and profiles in asymptomatically and symptomatically infected *G. pallidipes* flies determined. Thirty-eight host-encoded microRNAs (miRNAs) were present in both the asymptomatic and symptomatic fly profiles, while nine host miRNAs were expressed specifically in asymptomatic flies versus 10 in symptomatic flies. Of the shared 38 miRNAs, 15 were differentially expressed when comparing asymptomatic with symptomatic flies. The most up-regulated host miRNAs in symptomatic flies was predicted to target immune-related mRNAs of the host. Six GpSGHV-encoded miRNAs were identified, of which five of them were only in symptomatic flies. These virus-encoded miRNAs may not only target host immune genes but may also participate in viral immune evasion. This evidence of differential host miRNA profile in *Glossina* in symptomatic flies advances our understanding of the GpSGHV-*Glossina* interactions and provides potential new avenues, for instance by utilization of particular miRNA inhibitors or mimics to better manage GpSGHV infections in tsetse mass-rearing facilities, a prerequisite for successful SIT implementation.

## Introduction

The *Glossina pallidipes* salivary gland hypertrophy virus (GpSGHV; family *Hytrosaviridae*) is a large, rod-shaped dsDNA virus pathogenic to some species of tsetse flies (*Glossina* spp.) (Abd-Alla et al., 2010a). In the majority of tsetse fly species, GpSGHV exists predominantly in asymptomatic (covert) infection state, but on rare occasions, some unknown conditions can trigger the expression of overt symptomatic (overt) infections in some tsetse species such as *G. pallidipes* (Abd-Alla et al., 2010b; Kariithi et al., 2013). Asymptomatic (presumed latent) infection state has no apparent fitness cost to infected flies, while symptomatic infections are associated with reproductive dysfunctions that sometimes result in collapse of infected fly colonies (Abd-Alla et al., 2010b, 2011). Asymptomatically infected female *G. pallidipes* can vertically transmit the virus to their offspring through milk gland secretions or transovarially (Abd-Alla et al., 2010b). In tsetse mass rearing facilities, horizontal transmission of the virus mainly occurs during collective *in vitro* membrane feeding, whereby symptomatic flies release the virus via the saliva when taking a blood meal (Abd-Alla et al., 2011). Symptomatic infections in *G. pallidipes* are characterized by detectable salivary gland hypertrophy (SGH). The symptomatic infections also result in testicular degeneration in males and ovarian abnormalities in females, which leads to decreased fecundity of the colony (Abd-Alla et al., 2010b). Therefore, the occurrence of symptomatic GpSGHV in colonies of *G. pallidipes* makes colony maintenance challenging and drastically increase the risk of colony decline or even collapse (Abd-Alla et al., 2013).

Maintenance of healthy tsetse fly colonies is crucial for the application of the sterile insect technique (SIT) to manage tsetse fly populations and African trypanosomosis, the disease these flies transmit (Feldmann et al., 2005). SIT requires mass release of sterile males into the target wild insect population to mate with virgin wild females. These matings will produce no offspring in the target population, which will eventually decline as the population replacement rate is reduced (Knipling, 1959). Symptomatic virus outbreaks in mass-rearing facilities of tsetse species such as *G. pallidipes* are a serious impediment to the implementation of the SIT. This has stimulated research efforts to better understand virus-host interactions at the molecular level and to identify the parameters that determine whether GpSGHV infections become symptomatic or remain covert (Abd-Alla et al., 2010b). The RNA interference (RNAi) pathways, which are mediated by short interfering RNA (siRNA) and microRNA (miRNA), are known to modulate virus-host interactions in insects, thereby providing an antiviral defense (Van Rij, 2008). P-element induced wimpy (PIWI) testis in *Drosophila*-interacting RNAs (piRNAs) are a separate group of non-coding small RNAs of 25–30 nucleotides (nt) that have been shown to repress transposable elements and regulate cellular genes (Luo and Lu, 2017). The piRNAs have recently been shown to play a role in antiviral strategies in insects against arboviruses (Miesen et al., 2016).

MiRNAs are short (18–24 nt) non-coding RNAs that regulate host or pathogen gene expression post-transcriptionally by binding to complementary regions located mainly in the 3′ untranslated regions (3′-UTRs) of targeted mRNAs (Hussain and Asgari, 2010). The miRNAs regulate virus infection and other biological processes in animals, plants and insects (Skalsky and Cullen, 2010). For a number of dipteran insects, it has been shown that the miRNA expression profile changes during virus infection and in this way the expression level of host genes with a role in immunity can be modulated (Lucas and Raikhel, 2013). For instance, in the yellow fever mosquito, *Aedes aegypti*, the host miRNA aae-miR-374 enhanced dengue virus (DENV) infection, while another host miRNA aae-miR-2940 reduced replication of the virus (Zhang et al., 2013; Asgari, 2014). These findings indicate that miRNAs can either positively or negatively regulate the host defense to pathogen infection. Viruses may also encode miRNAs that target host cellular mRNAs and, in that way manipulate host gene expression and ensure effective virus proliferation. Virus-encoded miRNAs may target host or virus genes in order to maintain a latent infection state (Cullen, 2009; He et al., 2014). For instance, it has been reported that the DNA virus *Heliothis virescens* ascovirus (HvAV) encodes an miRNA, HvAV-miR-1, that targets its own DNA polymerase thereby inhibiting lytic virus infection and maintaining a persistent state of the virus (Hussain et al., 2008).

The role of miRNAs in virus-host interactions has been demonstrated in many insects, but there is limited information on how GpSGHV infection affects the miRNA profile in tsetse flies. The hypothesis is that GpSGHV alters both viral- and host-encoded miRNA profile in tsetse flies and that specific miRNAs may play a role in inducing or facilitating SGH in some cases and a latent infection state in other cases. The current study was designed to investigate the role of host and virus-encoded miRNAs during GpSGHV asymptomatic and symptomatic infection in the tsetse fly *G. pallidipes*. Deep sequencing of small RNA (sRNA) molecules was used to identify host and GpSGHV-encoded miRNAs and to determine whether these were differentially expressed in asymptomatically and symptomatically infected flies, or not. Furthermore, we predicted the mRNA targets of the differentially expressed host miRNAs and the GpSGHV-encoded miRNAs to investigate their potential roles during GpSGHV symptomatic infection. Finally, using inhibitors and miRNA mimics the functional significance of some miRNA was experimentally validated. This study presents important information on the interaction between GpSGHV and *G. pallidipes* miRNAs and provides potential avenues to further study the mechanisms of immune response during GpSGHV infections in tsetse flies.

## Materials and Methods

### Tsetse Flies, GpSGHV Preparation and Injection

The *G. pallidipes* flies used in this study were obtained from the colony maintained at the Joint FAO/IAEA Insect Pest Control Laboratory (IPCL), Seibersdorf, Austria. The flies were maintained in an environment-controlled insectary at 23 ± 1°C, 75–80% relative humidity, and a 12 h photo-phase. The flies were fed for 10–15 min, three times per week on defibrinated bovine blood using an *in vitro* membrane feeding system (Feldmann, 1994). The virus inoculum used was the Ugandan GpSGHV isolate prepared from one pair of salivary glands dissected from *G. pallidipes* flies showing overt SGH and homogenized in phosphate buffered saline (PBS) as described previously (Boucias et al., 2013). As a *G. pallidipes* colony free of overt SGH symptoms has been established in the IPCL, to obtain symptomatic flies, experimental flies were intra-hemocoelically injected with 2 μl of the virus inoculum, which was estimated to contain ∼10^6^ virus genome copies per μl of the virus suspension by quantitative polymerase chain reaction (qPCR) as described previously (Boucias et al., 2013). The progeny of these artificially infected mothers were used for the experiments that required symptomatically infected flies (confirmed microscopically during salivary gland dissections). A lack of a virus-free *G. pallidipes* flies at the IPCL colony precluded inclusion of a non-infected control group in the assays. Instead, flies of the same age as those of the above-described symptomatic fly group were directly sourced from the SGH-free colony and used for the asymptomatic (control) group.

### RNA Isolation, Small RNA Library Construction and Deep Sequencing

Prior to RNA extraction, flies were individually dissected to confirm their SGH status (symptomatic or asymptomatic). The virus presence was not confirmed by PCR since the flies were progeny of GpSGHV-injected *G. pallidipes* parents, which are known to produce only asymptomatically or symptomatically infected progeny (Boucias et al., 2013). Total RNA was extracted using Trizol (Invitrogen) from 10-day old flies, eight flies from each group of asymptomatically or symptomatically infected flies. To prepare sRNAs, the extracted total RNA was purified from denaturing polyacrylamide gel and sequentially ligated to the adapters for next generating sequencing (NGS) according to the manufacturer’s instructions (Illumina Inc.) before sequencing. The RNA quality was assessed on an Agilent 2100 Bioanalyzer (Agilent Technologies) (Masotti and Preckel, 2006). Two biological replicates from each group of barcoded libraries were then sequenced on Illumina Genome Analyzer Miseq (1 × 50 run) for 50 cycles to produce 3–5 million reads per library. Raw sequencing data have been submitted to the National Center for Biotechnology Information under the accession number SRP139935.

### Small RNA Analysis

The CLC genomic workbench version 11.0.1^1^ was used to remove adapter sequences and low-quality sequence reads from the datasets by applying a quality trimming cut off score of 0.05. The sequence reads without the 3′ adapters were discarded from the libraries. The small RNA tool of the CLC genomic workbench was used to extract and count unique sRNA reads. Clean sequence reads with lengths ranging from 18 to 30 nt were mapped against the *G. pallidipes* (GCA_000688715.1) genome found on the VectorBase database^2^ (Giraldo-Calderón et al., 2014) and against the genome of the Ugandan GpSGHV isolate (Accession Number: EF568108), allowing only a mismatch, insertion and deletion costs of 2, 3, and 3, respectively. The Rfam (RNA families) database^3^ was used to remove ribosomal RNAs (rRNAs), transfer RNAs (tRNAs), small nuclear ribonucleic acid RNAs (snRNAs) and repeats from the sRNA sequences (Kalvari et al., 2017). The remaining sequence reads were uploaded onto the CLC Genomic Workbench “annotate and merge counts tool” to search for conserved precursors and mature miRNAs using insect miRNA sequences found in the miRBase 22.0 (Griffiths-Jones et al., 2007) as reference. Only the perfectly or near-perfectly (1–2 mismatches) matching sequences were considered to represent conserved miRNAs.

To identify the GpSGHV-encoded miRNAs, the unique reads that mapped to the GpSGHV genome were combined with 150 nt either upstream or downstream from their position on the virus genome. The RNA secondary structures of the predicted pre-miRNA hairpins were analyzed using RNAfold^4^ (Lorenz et al., 2011). The hairpins were considered pre-miRNA if a mature miRNA was present in the arm of the hairpin precursor and the secondary structure was stable with low free energy of hybridization.

The GpSGHV-encoded miRNAs identified from the sRNA NGS data were then compared to the GpSGHV miRNA hairpins predicted by the VMir software using the GpSGHV genome (Grundhoff, 2011). The miRNA hairpin prediction by VMir was initially performed with the program default settings and later the values for minimum score and window counts were adjusted to 115 and 30, respectively, to increase the stringency for the hairpin selection as previously described and optimized (Grundhoff, 2011; Hussain et al., 2011).

### miRNA Differential Expression

The miRNA expression for each independent biological replicate was normalized on the CLC genomic workbench using the option “by totals,” which applies tag (number of copies of the sRNAs) per million total RNA reads (TPM). The normalized mean values of the two replicates were used to compare miRNA abundance or expression in asymptomatic and symptomatic libraries. Because of the low abundance of some of the identified miRNAs, only the miRNAs with more than 10 raw reads in the libraries were included in the differential expression analysis. Changes in miRNA expression in the symptomatic versus asymptomatic flies were considered significant when their *P*-values were below 0.05. The final fold change values were given in log_2_ scale and the miRNAs with log_2_-fold change (log_2_FC) higher than 0.2 or less than −0.1 were designated as up-regulated and down-regulated, respectively, in symptomatic flies.

### Putative Viral and Host miRNA Target Identification and Functional Analysis

RNA22^5^ and RNAhybrid^6^ software packages were used to predict the putative host and virus gene targets of the differentially expressed host- and GpSGHV-encoded miRNAs (Krüger and Rehmsmeier, 2006; Miranda et al., 2006). The miRNA target prediction with the RNAhybrid was performed using default settings with energy threshold set to −20 kcal/mol. The RNA22 software *P*-value was set to 0.05 and a minimum free energy (mfe) of <-12.0 kcal/mol and the remaining parameters were set to default. Only putative target genes that were predicted by both software packages were selected for further analysis. Gene Ontology (GO) enrichment and pathway analysis of the miRNA-targeted genes was performed using Blast2GO version 5.1.13^7^ (Conesa et al., 2005). To select only the putative immune related genes and their immune pathways, the miRNA targeted genes were further analyzed by protein blast (BLASTp; *e*-value ≤ 10^−2^) on the Insect Innate Immunity Database (IIID)^8^ (Brucker et al., 2012). Based on the *P*-values and the mfe values (lowest *P*-value and minimum mfe) for miRNA-mRNA interaction, the top 10 immune related genes targeted by the regulated host miRNAs and the GpSGHV-encoded miRNAs were used to generate host miRNA, GpSGHV-encoded miRNA and host mRNA interaction networks using Cytoscape^9^ (Shannon et al., 2003).

### RT-qPCR of miRNAs and Their Predicted Putative mRNA Targets

To validate the differentially expressed miRNAs during GpSGHV infections, reverse transcription qPCR (RT-qPCR) was used. Total RNA was extracted using Trizol as described above from eight asymptomatically and eight symptomatically infected flies. Complementary DNA (cDNA) was synthesized using the miSCRIPT II RT kit (Qiagen) using the Hiflex buffer to ensure cDNA synthesis of both miRNAs and mRNA molecules. The qPCR was performed using the miScript SYBR Green PCR kit (Qiagen), which includes an miRNA universal reverse primer. The forward primer in the reactions for miRNA quantification was derived from each of the specific miRNA sequences investigated in this study (see details in **Supplementary Table 1**). The PCR program used to quantify the miRNAs was; 95°C for 15 min, followed by forty cycles of 94°C for 15 s, 55°C for 30 s, and 70°C for 30 s. To determine the impact of the virus regulated host miRNAs on their mRNA transcript levels, the expression levels of the selected top 10 immune related genes targeted by the miRNAs was assessed in the same asymptomatic and symptomatic flies using the primers listed in **Supplementary Table 1**. The virus infection level was estimated by quantifying the expression of the conserved GpSGHV *odv-e66* gene whose transcript levels have been correlated to the total virus copy numbers (Abd-Alla et al., 2009) using the primers in **Supplementary Table 1**. The PCR program to quantify the *odv-e66* and the selected immune genes was; 95°C for 15 min, followed by forty cycles of 94°C for 15 s, 60°C for 30 s, and 70°C for 30 s. Two technical replicates were included for each reaction and all the target gene expressions were normalized to tsetse *β-tubulin* gene expression using previously described primers (Caljon et al., 2009).

### Inhibition of miR-184-3p in *G. pallidipes*

To investigate the role of the most up-regulated host miRNA with high abudance in symptomatically infected flies compared to the asymptomatic individuals (i.e., miR-184-3p), an inhibitor and a mimic of this miRNA were synthesized (Thermo Fisher company; Waltham, MA, United States) and subsequently injected into the flies. The details of the inhibitor and the mimic sequences are included in **Supplementary Table 1**. Prior to the above-mentioned injections, teneral (24 h post eclosion; un-fed) adult flies were anaesthetized on ice, and then injected in the thorax with 10 pmol of either the inhibitor or the mimic or RNase free water (40 females and 40 males per group). Two days post injection with the inhibitor, mimic, or RNase-free water, half of the flies from each group (20 females and 20 males) were injected with PBS (control), and the other half of the flies were injected with 2 μl of the virus inoculum as described above. The ability of GpSGHV-injected female parents to induce symptomatic infections in the progeny depends on the increase in virus titre in parents (Boucias et al., 2013). Therefore, in our study, samples from the parental generation (i.e., 3 females and 3 males), which were collected at zero and 21 days post PBS/virus injection followed by RNA extraction and cDNA synthesis were analyzed using miSCRIPT II RT kit as described above. To validate the success of the inhibitor and the mimic of miR-184-3p, the expression levels of miR-184-3p in the collected samples were assessed using the miScript SYBR Green PCR kit. The effect of injecting miR-184-3p inhibitor or mimic on virus infection was determined by quantifying the GpSGHV *odv-e66* gene expression on the day of injection and 21 days post PBS/virus injection. The expression levels were normalized to *β-tubulin* gene as described in the previous section.

### Statistical Analysis

The RT-qPCR results were representatives of three biological replicates each with two technical replicates. Statistical tests were performed with RStudio v1.0.143 (Allaire, 2012) [R v3.4.0 (R Core Team, 2017)] using the packages lattice v0.20-35 (Sarkar, 2008) and MASS v7.3.47 (Venables and Ripley, 2013) and visualized using ggplot2 v2.2.1 (Wickham, 2009). The data were normalized and transformed using the Box-Cox routine. The data were log transformed if the 95% confidence interval of the lambda value included zero and transformed with (x^λ^ − 1)/λ in other cases. Student’s *t*-tests were used for the comparison of RT-qPCR data.

## Results

### Deep Sequencing Data and Small RNA Profiles

**FIGURE 1:**
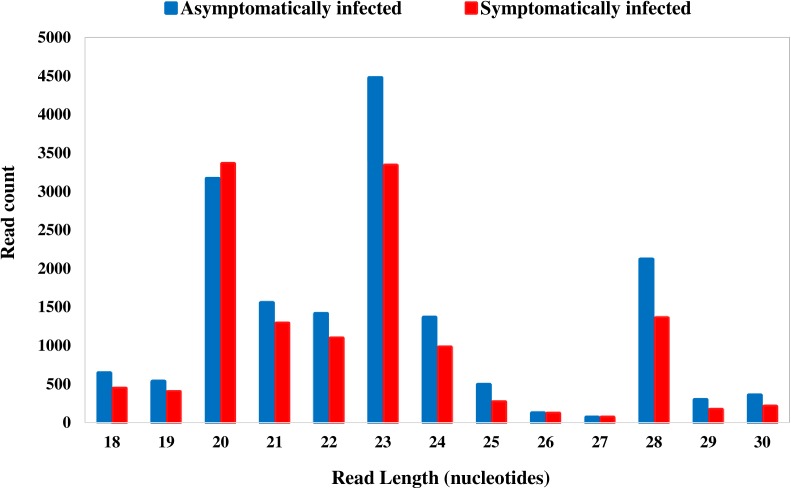
Length distribution of reads mapped onto the *G. pallidipes* genome from asymptomatically (blue bar) and symptomatically (red bar) infected flies.

The Illumina deep sequencing of the sRNAs to profile the miRNA in the libraries of the asymptomatically and symptomatically infected *G. pallidipes* flies produced a total of 6.3 and 4.8 million reads, respectively, from the combined biological replicates. Although the total raw reads differed considerably between the two independent biological replicates in each group (**Table 1**), further analyses with independent normalization of the data revealed similar results. Removal of adaptors, contaminants and low-quality reads resulted in ∼20,166 and ∼16,309 reads from the asymptomatic and symptomatic libraries, respectively. Of these, 16,422 and 13,077 clean reads from the asymptomatic and symptomatic libraries, respectively, were mapped onto the *G. pallidipes* genome (**Table 1**). A summary of the length distribution of the clean reads that mapped onto the *G. pallidipes* genome is shown in **Figure 1**. The number of sRNAs over length distribution was generally less in symptomatic libraries compared to the asymptomatic libraries. Both libraries showed a peak at 20–23 nt, which may represent the class of miRNA or siRNAs, and another frequency peak was observed at a length of 28 nt, which may represent the piRNAs. Mapping of the clean reads onto the GpSGHV genome resulted in three viral sRNA from the asymptomatic compared to 53 sRNAs from symptomatic fly libraries (see details in **Supplementary Table 2**).

**Table 1:** A summary of sRNA from GpSGHV asymptomatically and symptomatically infected flies.

Class of RNA	Asymptomatic library R1	Symptomatic library R1	Asymptomatic library R2	Symptomatic library R2	Asymptomatic library (total)	Symptomatic library (total)
Raw reads	1,735,077	1,167,975	4,610,021	3,699,655	6,345,098	4,867,630
Reads after quality filtering	4,567	4,456	15,599	11,853	20,166	16,309
Reads mapped to *G. pallidipes* genome	3,624	3,521	12,798	9,556	16,422	13,077
Reads mapped to SGHV genome	0	13	3	40	3	53
*G. pallidipes* miRNAs reads	290	271	1,385	936	1,675	1,207
GpSGHV miRNAs reads	0	0	1	6	1	6
Unmapped reads	943	935	757	526	1,700	1,461
rRNA	555	518	390	302	945	820

### Identification of Host- and Virus- Encoded miRNAs

Analyses of the sRNAs in *G. pallidipes* revealed that 1,675 and 1,207 reads putatively coded for host miRNAs in asymptomatic and symptomatic flies, respectively (**Supplementary Table 3**). A total of 57 host miRNAs were identified, which were named with sequential numbers following their gene family as designated in the miRBase. Of the 57 host miRNAs, 38 miRNAs were expressed in both asymptomatic and symptomatic flies, while nine and 10 of the miRNAs were found only in asymptomatic and symptomatic flies, respectively. Notably, six of the 57 identified host miRNAs (i.e., miR-1-3p, miR-184-3p, miR-263-5p, miR-277-3p, miR-283-5p, and miR-8-3p) were found to be conserved in five insect species (*Ae. aegypti*, *Anopheles gambiae*, *Bombyx mori*, *Drosophila melanogaster*, and *Apis mellifera*) from the miRBase (data not shown).

**FIGURE 2:**
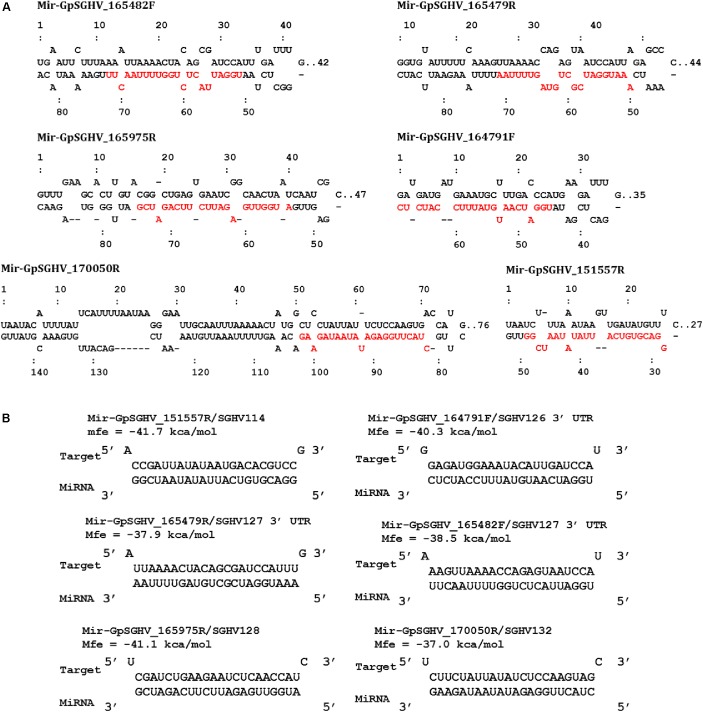
GpSGHV encoded miRNAs identified from the NGS data. **(A)** Secondary structures of the GpSGHV pre-miRNAs named according to their position and orientation on the GpSGHV-UGA genome (accession number: EF568108) mature miRNA sequences are presented in red. **(B)** Interaction of mature GpSGHV encoded miRNA sequences and their possible virus mRNA targets.

Based on the criteria for pre-miRNA prediction (i.e., the presence of mature miRNA and mfe <−20 kcal/mol) and the secondary structure analysis, six putative GpSGHV-encoded miRNAs were identified from the 60 sRNA reads that mapped onto the GpSGHV genome from the symptomatic library. The identified GpSGHV-encoded miRNAs were named according to their position and orientation on the GpSGHV-Uga genome. These six putative GpSGHV-encoded miRNAs were mir-GpSGHV_164791F, mir-GpSGHV_170050R, mir-GpSGHV_165482F, mir-GpSGHV_165479R, mir-GpSGHV_151557R, and mir-GpSGHV_165975R (**Table 2**). Analysis of the secondary structures analysis of the GpSGHV-encoded pre-miRNAs revealed the 3′ overhangs associated with the Dicer/Drosha-mediated processing and the mature miRNA sequences (**Figure 2A**). Since most virus-encoded miRNAs appeared to be localized antisense to their viral transcripts, which could be the obvious potential targets (Sullivan et al., 2005), the possible GpSGHV-encoded miRNAs interacting with the respective GpSGHV transcripts were identified (**Figure 2B**). From the three sRNA reads that mapped onto the GpSGHV genome from the asymptomatic library, only one read was identified as mir-GpSGHV_170050R; this putative viral miRNA was also present in the symptomatic sRNA library.

**Table 2:** VMir predicted and sequenced GpSGHV pre-miRNA and their characteristics.

MiRNA name	Pre-miRNA length	Pre-miRNA mfe (kcal/mol)	Strand	Position in GpSGHV genome	VMir score	Mature miRNA length	Mature miRNA location	Mature miRNA sequence (5′-3′)
Mir-GpSGHV_170050R	145	−41.60	Reverse	170050–170194	254.9	22	3p	CTACTTGGAGATATAATAGAAG
Mir-GpSGHV_165479R	87	−22.70	Reverse	165479–165565	217.9	22	3p	AAATGGATCGCTGTAGTTTTAA
Mir-GpSGHV_165481F	83	−21.0	Forward	165481–165564	214.1	22	3p	TGGATTACTCTGGTTTTAACTT
Mir-GpSGHV_165975R	87	−23.60	Reverse	165975–166062	180.7	22	3p	ATGGTTGAGATTCTTCAGATCG
Mir-GpSGHV_164791F	66	−19.0	Forward	164791–164857	137.4	22	3p	TGGATCAATGTATTTCCATCTC
Mir-GpSGHV_151557R	55	−9.30	Reverse	151532–151583	130	22	3p	GGACGTGTCATTATATAATCGG

**FIGURE 3:**
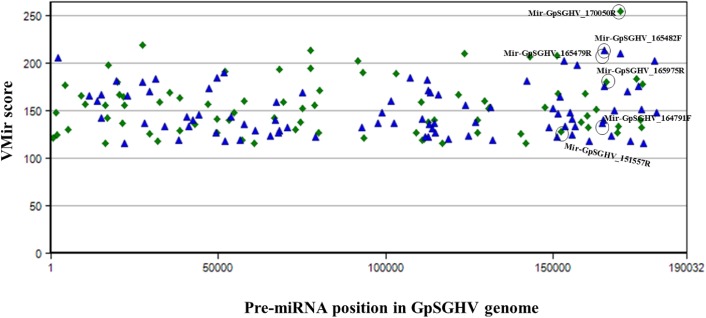
Distribution of predicted pre-miRNA hairpins on the GpSGHV-UGA genome. The hairpins are plotted according to their location on GpSGHV-UGA genome and their VMir score. Only the hairpins with VMir score of above 115 and that can fold in 35 or more windows are plotted. The green diamonds indicate the hairpins on the reverse orientation while the blue triangles indicate the hairpins on the forward orientation. The hairpins corresponding to the miRNAs identified by NGS are circled in black.

The identified viral miRNAs from the sRNA libraries were compared to the VMir predicted pre-miRNA hairpins, whereby the GpSGHV genome was supplied to the VMir program, and the initial pre-miRNA hairpin search (without filtering) detected a total of 2,328 main hairpins (MHPs). After filtering with settings of 115 and 35 as the values for minimum scores and window counts, respectively, 167 pre-miRNA hairpins were selected. Notably, four of the six GpSGHV-encoded miRNAs identified from the Illumina sequencing library were among the pre-miRNA hairpins that were predicted with high scores (**Figure 3**). The locations of the predicted pre-miRNA hairpins on the GpSGHV genome and their VMir scores are also presented in **Figure 3**, showing that the high-scoring pre-miRNA hairpins are located between nucleotides 143,000 and 180,000 of the GpSGHV genome. This is similar to the locations of the experimentally obtained; Illumina sequenced viral miRNAs (circled in green in **Figure 3**).

### Differential Expression of miRNAs in Asymptomatic and Symptomatic Flies

**FIGURE 4:**
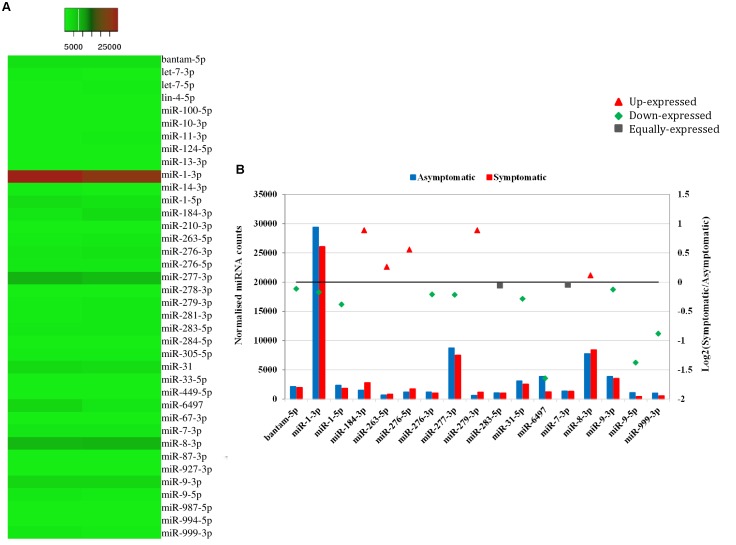
Differential expression of miRNAs in asymptomatic and symptomatic flies. **(A)** Heat map of the co-expressed host miRNAs. The highly expressed miRNAs are shown in red and the low expressed in green with absolute signal intensity ranging from 1 to 25000. **(B)** Expression analysis of the host miRNAs with more than 10 raw reads. The horizontal axis, the left vertical axis and the right vertical axis indicate the miRNA, the normalized miRNA expression values and the log_2_ fold change of the miRNA, respectively. The log_2_ fold change (Log2FC) of the up-regulated miRNAs are shown in red, down-regulated in green and equally expressed in black.

Heat mapping of the above-mentioned 38 host miRNAs identified in both libraries of *G. pallidipes* revealed different expression patterns between the asymptomatic and symptomatic flies (**Figure 4A**). After excluding the host miRNAs with less than 10 raw reads from the asymptomatic and symptomatic libraries, we analyzed the differential expression of the 17 remaining host miRNAs. Based on the log_2_FC (>0.1 or <-0.1), 15 miRNAs were considered as differentially expressed, of which 10 were down-regulated and five were up-regulated in the symptomatic flies. The up-regulated (enriched in symptomatic flies compared to their asymptomatic counterparts) miRNAs were miR-184-3p, miR-279-3p, miR-276-5p, miR-263-5p, and miR-8-3p with log_2_FC values of 0.9, 0.9, 0.6, 0.3, and 0.1, respectively. Among the down-regulated (depleted in symptomatic flies) miRNAs with maximum log_2_FC were miR-6497, miR-9-5p, miR-999-3p, miR-1-5p, and miR-31-5p with log_2_FC of −1.6, −1.4, −0.9, −0.4, and −0.3, respectively (**Figure 4B**). There was no change in the expression (0.0 log_2_FC) of miR-283-5p and miR-7-3p, and hence these were considered equally expressed in both asymptomatic and symptomatic flies (**Figure 4B**).

### Validation of Differentially Expressed miRNAs in *G. pallidipes* by RT-qPCR

**FIGURE 5:**
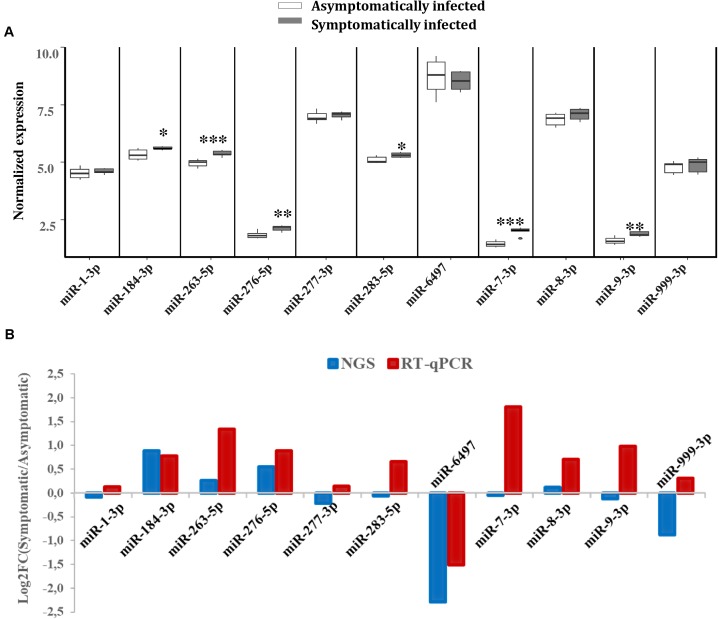
Differential expression of *G. pallidipes* miRNAs upon symptomatic infection. **(A)** Reverse transcription qPCR data analysis of selected host miRNAs: Expression profiles of miRNAs in asymptomatic and symptomatic *G. pallidipes*. Error bars show the standard deviation from the replicates. Asterisks indicate the statistical significance: ^∗∗∗^*P* < 0.001, ^∗∗^*P* < 0.01, ^∗^*P* < 0.05. **(B)** The graph shows a comparison of Log2 fold changes of *G. pallidipes* miRNAs based on NGS and Reverse transcription qPCR analysis.

To validate the expression levels of some of the miRNAs that were differentially expressed according to our NGS data, RT-qPCR analysis was performed on a separate pool of asymptomatic and symptomatic flies to further quantify these miRNAs. The RT-qPCR results showed that the differences in the expression levels of the analyzed miRNAs when comparing asymptomatic and symptomatic flies was mostly consistent with the earlier observed differences in the NGS data. For instance, the up-regulated miRNAs miR-184-3p, miR-276-5p, miR-263-5p, and miR-8-3p, according to the NGS analysis, were up-regulated during symptomatic infection by RT-qPCR approach. The miRNAs that were considered down-regulated (e.g., miR-6497, miR-1-3p, miR-277-3p, and miR-999-3p) based on the NGS data, showed no significant change in their expression when using the RT-qPCR approach (**Figure 5A**). The only inconsistency was observed for the equally expressed miRNAs (miR-283-5p and miR-7-3p) and miR-9-3p (slighlty down-regulated) in symptomatic flies according to the NGS data, but showed up-regulation with the RT-qPCR analysis approach (**Figure 5B**).

### Prediction of mRNA Targets for the Differentially Expressed Host miRNAs

**FIGURE 6:**
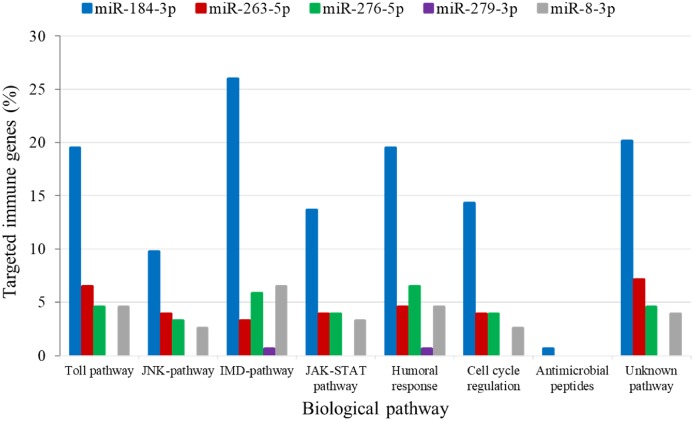
Biological immune pathways of the immune related genes targeted by the up-regulated miRNAs; miR-184-3p (blue bars), miR-263-5p (red bars), miR-276-5p (green bars), miR-279-3p (purple bars), and miR-8-3p (gray bars). The percentages of the immune genes regulated by each miRNA are shown in different colors for each immune pathway.

**FIGURE 7:**
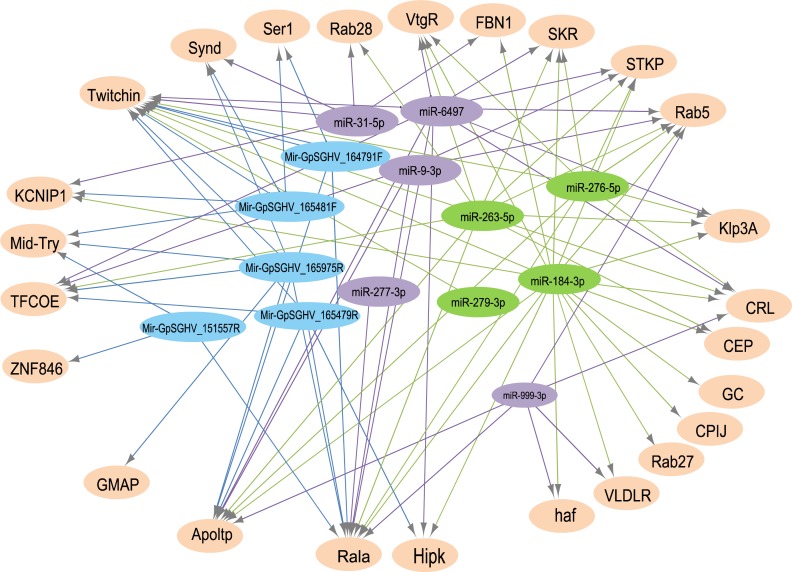
A network of interactions between up-regulated (in green) and down-regulated (in purple) host miRNAs, GpSGHV-encoded miRNAs (in blue) and some selected target genes (light orange). The network was visualized using Cytoscape with miRNAs as the source nodes and the mRNA as the targets. Abbreviations: *KCNIP1*, Kv channel-interacting protein 1; *ZNF846*, zinc finger protein 846; *CEP*, centrosomal protein; *GC*, guanylyl cyclase; *FBN1*, fibrillin-1; *Klp3A*, kinesin-like protein at 3A; *GMAP*, golgi microtubule-associated protein; *Rala*, Ras-related protein Ral-a; *VLDLR*, very low-density lipoprotein receptor domain class A; *CPIJ*, cuticular protein 50Cb; *SKR*, serine/threonine-protein kinase receptor; *Hipk*, homeodomain interacting protein kinase; *TFCOE*, transcription factor collier; *Rab27*, Ras-related protein Rab-27A; *CRL*, cytokine receptor-like; *Synd*, Syndapin; *Ser1*, serine protease 1; *Apoltp*, apolipoprotein lipid transfer particle; *Mid-Try*, Midgut trypsin; *STKP*, protein-serine/threonine kinase; *VtgR*, vitellogenin receptor; *haf*, hattifattener.

The analysis for potential mRNA targets of the 15 differentially expressed miRNAs as identified by NGS revealed that the miRNAs up-regulated in symptomatic flies potentially targeted about 715 putative host mRNAs, compared to 757 genes that were potentially targeted by the down-regulated miRNAs (**Supplementary Tables 4A,B**). Of the 715 putative genes targeted by the miRNAs up-regulated in symptomatic flies, 154 were immune-related such as Ras-related protein-27 (*Rab27*), homeodomain interacting protein kinase (*Hipk*) and apolipoprotein lipid transfer particle (*Apoltp*) (**Supplementary Table 4C**). The blast search using the IIID software revealed that the immune-related genes could be involved in various immune signaling pathways, mostly in immune deficiency (Imd), humoral response and Toll pathways, and some of these genes were involved in multiple immune pathways. Approximately 70% (108/154) of these immune genes targeted by the up-regulated miRNAs were targeted by miR-184-3p, which was among the most highly up-regulated miRNA (0.9 log_2_FC) in symptomatic flies (**Figure 6**) and one of the miRNAs confirmed to be up-regulated with the qRT-PCR experiment (**Figures 5A,B**). Notably, only one of these 154 immune-related genes was a potential target of miR-279-3p, the other of the most up-regulated miRNA (0.9 log_2_FC) in symptomatic flies. Additionally, about 30% (35/108) of the miR-184-3p immune targeted genes were also targeted by the identified GpSGHV-encoded miRNAs (**Supplementary Table 4D**). The possible interactions between the host regulated miRNAs, the GpSGHV-encoded miRNAs and a selection of their targeted genes (as determined using Cytoscape) are presented in the network in **Figure 7** (**Supplementary Table 4E**).

**FIGURE 8:**
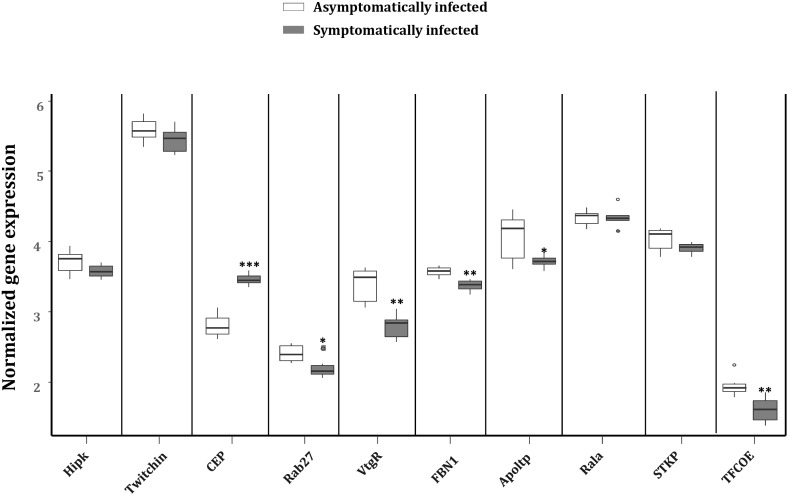
Reverse transcription qPCR data analysis of selected immune target genes in asymptomatically and symptomatically infected *G. pallidipes*. Error bars show the standard deviation from the replicates. Asterisks indicate the statistical significance: ^∗∗∗^*P* < 0.001, ^∗∗^*P* < 0.01, ^∗^*P* < 0.05. Abbreviations: *CEP*, centrosomal protein; *FBN1*, fibrillin-1; *Rala*, Ras-related protein Ral-a; *Hipk*, homeodomain interacting protein kinase; *Rab27*, Ras-related protein Rab-27A; *Apoltp*, apolipoprotein lipid transfer particle; *TFCOE*, transcription factor collier; *STKP*, protein-serine/threonine kinase; *VtgR*, vitellogenin receptor.

To explore the impact of the regulated miRNAs on host immunity, RT-qPCR expression analysis of 10 potentially targeted immune genes [Centrosomal protein (*CEP*), fibrillin-1 (*FBN1*), Ras-related protein (*Ral-a*), *Hipk*, *Rab27*, *Apoltp*, Transcription factor collier (*TFCOE*), Protein-serine/threonine kinase (*STKP), Twitchin* and Vitellogenin receptor (*VtgR*)] was performed on asymptomatically and symptomatically infected flies (**Table 3**). The analysis showed that five of these immune genes (*Fibrillin-1*, *Rab27*, *VtgR*, *TFCOE*, and *Apoltp*) were down-regulated, while only the *CEP* was up-regulated in symptomatic flies. There was no difference in expression levels of the genes encoding *Hipk*, *Twitchin*, *STKP* and *Ral-a* (**Figure 8**).

**Table 3:** The top 10 predicted targets of the differentially expressed host and GpSGHV-encoded miRNAs.

Target gene (VectoBase ID)	Gene name	miRNA	Molecular function/biological process
GPAI025158	Homeodomain interacting protein kinase (Hipk)	miR-184-3p	ATP binding, protein kinase activity. Plays a role in cell proliferation and development
		Mir-GpSGHV_165479R	
		miR-6497	
GPAI030501	Ras-related protein Rab-27	miR-184-3p	GTPase activity Source, GTP binding. Involved in exocytosis and phagocytosis
GPAI014544	Fibrillin-1 (FBN1)	miR-184-3p	Calcium ion binding. Involved in cell communication
		miR-31-5p	
GPAI038987	Protein-serine/threonine kinase (STKP)	miR-6497	ATP binding, G-protein coupled receptor kinase activity. Involved in regulation of innate immune response and oogenesis
		miR-9-3p	
		miR-263-5p	
		miR-184-3p	
		miR-276-5p	
GPAI034557	Apolipoprotein lipid transfer particle (Apoltp)	miR-184-3p	Lipid transporter activity, lipoprotein particle receptor binding. Provides the major yolk proteins during vitellogenesis
		miR-999-3p	
		miR-6497	
		miR-276-5p	
		miR-263-5p	
		miR-9-3p	
		Mir-GpSGHV_165479R	
		Mir-GpSGHV_165975R	
		Mir-GpSGHV_164791F	
GPAI025990	Transcription factor collier (TFCOE)	miR-6497	DNA binding, metal ion binding. Involved in development
		miR-9-3p	
		miR-263-5p	
		Mir-GpSGHV_165975R	
		Mir-GpSGHV_165479R	
GPAI001218	Twitchin	miR-6497	ATP binding, protein kinase activity. Mesoderm development
		miR-276-5p	
		miR-263-5p	
		miR-184-3p	
		miR-9-3p	
		miR-31-5p	
		miR-279-3p	
		Mir-GpSGHV_165975R	
		Mir-GpSGHV_164791F	
		Mir-GpSGHV_165482F	
		Mir-GpSGHV_165479R	
GPAI015640	Ras-related protein Ral-a (Rala)	miR-184-3p	GTPase activity, GTP binding. Innate immune response and signal transduction
		miR-277-3p	
		miR-999-3p	
		miR-6497	
		miR-263-5p	
		miR-276-5p	
		miR-9-3p	
		Mir-GpSGHV_151557R	
		Mir-GpSGHV_165975R	
		Mir-GpSGHV_164791F	
		Mir-GpSGHV_165479R	
GPAI042543	Vitellogenin receptor (VtgR)	miR-6497	Calcium ion binding. Involved in uptake of vitellogenin by endocytosis during oogenesis
		miR-184-3p	
		miR-276-5p	
		miR-263-5p	
GPAI007448	Centrosomal protein (CEP)	miR-184-3p	Centriole-centriole cohesion Source, centriole replication. Involved in spermatogenesis
		miR-263-5p	

### Role of miR-184-3p During GpSGHV Infection

**FIGURE 9:**
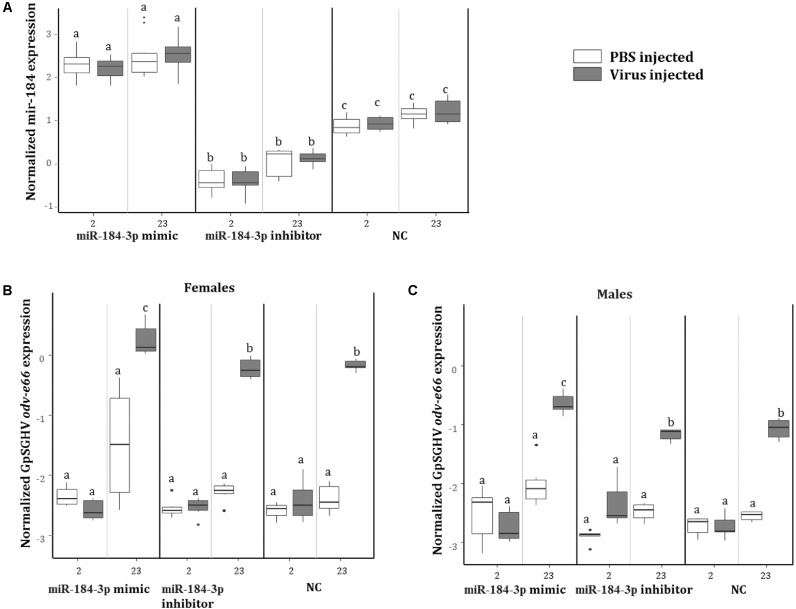
Impact of miR-184-3p inhibitor or mimic injection on GpSGHV infection. **(A)** Expression analysis of miR-184-3p, 2 and 23 days post injection of mimic or inhibitor in *G. pallidipes*. **(B)** Expression levels of GpSGHV odv-e66 gene following miR-184-3p inhibitor or mimic injection in *G. pallidipes* females and **(C)** males compared to the RNase-free water injected flies (NC). Error bars show the standard deviation from the replicates. The expression results marked with the same lower-case letter do not differ at the 0.05 level.

To investigate the role of miR-184-3p, the most up-regulated miRNA in symptomatic flies (0.9 log_2_FC) that potentially targets most of the immune genes, during GpSGHV infection, miR-184-3p inhibitor and mimic sequences were designed and injected together with GpSGHV into *G. pallidipes*. A significant up-regulation and down-regulation of the expression of miR-184-3p was observed in the flies injected with the miR-184-3p mimic and inhibitor, respectively (**Figure 9A**). Injection of the miR-184-3p mimic showed a significant increase in GpSGHV *odv-e66* transcript levels, signaling up-regulated GpSGHV expression, while miR-184-3p inhibition had no impact on GpSGHV *odv-e66* transcript levels levels in both *G. pallidipes* females and males (**Figures 9B,C**).

## Discussion

In insects both host and virus-encoded miRNAs have been shown to regulate cellular responses, including immune responses, during virus infections (Asgari, 2014). Virus-encoded miRNAs may function just as cellular miRNAs and inhibit the translation of cellular mRNAs through direct interaction with its target mRNA, mediated by partial complementarity (Cullen, 2009; Kincaid and Sullivan, 2012). In addition, virus-encoded miRNAs can regulate virus encoded genes, especially genes that are involved in regulation of virus replication, and as such may control the latent and lytic infection stages by fully or partially aligning antisense to a target viral mRNA (Kincaid and Sullivan, 2012; He et al., 2014; Asgari, 2015). The molecular mechanisms of interactions between tsetse flies and GpSGHV are poorly understood, making it difficult to define the factors that determine the switch from asymptomatic to symptomatic infection. In this study, we aimed to identify the host and virus-encoded miRNAs that are expressed during GpSGHV infection in *G. pallidipes* by the Illumina sRNA sequencing approach. The elucidation of the role of miRNAs in GpSGHV-tsetse interactions may open avenues to manage virus replication or transmission in tsetse-mass rearing facilities, which would facilitate the implementation of the SIT as part of area-wide integrated pest management (AW-IPM) programs in the fight against tsetse and trypanosomosis.

Approximately 6.3 million sRNA reads were obtained from asymptomatically infected flies versus 4.8 million reads from symptomatically infected individuals, which may imply a depletion of sRNA synthesis and perhaps a reduced efficiency of the miRNA pathway during symptomatic infections. About 80% of the obtained total sRNA clean reads were mapped onto the genome of *G. pallidipes*. Most of the identified host miRNAs were also found to be conserved among other insects such as *Ae. aegypti* and *D. melanogaster* as predicted in the insect miRBase database. Among these miRNAs, miR-1-3p, miR-277-3p, and miR-8-3p were highly expressed in both asymptomatic and symptomatic flies suggesting that they may have potential roles in regulating gene expression in *G. pallidipes* in general. For instance, miR-1-3p is known to regulate muscle cell differentiation and development, respectively, in *Drosophila* (Kwon et al., 2005). We also found that 15 host miRNAs were differentially expressed in asymptomatically and symptomatically infected flies. According to the NGS data sets, miR-184-3p and miR-6497 were the most up- and down-regulated miRNAs, respectively, in symptomatic flies. MiR-184-3p has been reported to play a role during virus infections in mosquitoes (Maharaj et al., 2015) where it was up-regulated in *Ae. albopictus* mosquitoes infected with the chikungunya virus (CHIKV) and dengue virus (DENV) (Liu et al., 2015). MiR-184-3p has also been reported to be up-regulated in baculovirus-infected *Spodoptera frugiperda* cells (Mehrabadi et al., 2013). It is therefore possible that miR-184-3p is an immune-associated miRNA in *G. pallidipes*; miR-184-3p has been reported to regulate the phagocytosis and phenoloxidase pathways in shrimps (Yang et al., 2012). In addition, miR-184-3p has also been implicated in multiple roles in *Drosophila* where it regulates oogenesis and early embryogenesis (Iovino et al., 2009). Thus, the up-regulation of miR-184-3p in symptomatically infected *G. pallidipes* tsetse flies may hint at clues to the mechanism of GpSGHV-induced sterility.

The validation of the NGS differentially expressed miRNAs using RT-qPCR confirmed the expression of the up-regulated miRNAs (miR-184-3p miR-276-5p, miR-263-5p, and miR-8-3p) in symptomatic flies. Some inconsistencies were noted in the down-regulated and equally expressed miRNAs according to our NGS data, which showed no significant change in their expression or up-regulated according to RT-qPCR, respectively. Although different pools of asymptomatic and symptomatic flies were used for the analysis in the two approaches, these inconsistencies were unexpected as both approaches are considered highly sensitive and accurate. Nevertheless, such inconsistencies between RT-qPCR and NGS approaches for miRNA expression analysis are not uncommon as they have been observed in previous studies (Liu et al., 2015; Saldaña et al., 2017). Based on the *P*-values (*P* < 0.001) of the RT-qPCR analysis; miR-263-5p was the most up-regulated miRNA whose modulation by virus infections has been reported in CHIKV and DENV-infected *Ae. albopictus* (Liu et al., 2015; Maharaj et al., 2015). In CHIKV-infected *Ae. aegypti*, miR-263-5p was not only highly expressed but also up-regulated (Maharaj et al., 2015). This finding implies that miR-263-5p (in addition to miR-184-3p) may play certain roles during GpSGHV infections in *G. pallidipes*.

In addition to the identification of host miRNAs, we also identified six GpSGHV-encoded miRNAs that may potentially be involved in regulating GpSGHV infections. Four of these GpSGHV-encoded miRNAs were among the viral miRNA hairpins with high scores as predicted by VMir, a program that applies a low stringency prediction method by sliding a 500-nt window and utilizes RNAfold to analyze the RNA structure (Grundhoff, 2011). Additionally, these GpSGHV-encoded miRNAs have also been identified previously using sRNAloop (Garcia-Maruniak et al., 2009), a program that predicts pre-miRNAs based on sequence structure and thermodynamic analyses (Grad et al., 2003). These virus encoded-miRNAs may have potential roles during GpSGHV symptomatic infections, possibly to prolong the lifespan of the infected cells by targeting and suppressing pro-apoptotic host genes as reported, for instance, for an Epstein Barr virus (EBV)-encoded miRNA (miR-BART5) (Marquitz et al., 2011). The virus-encoded miRNAs can also assist in immune response evasion by negatively regulating early viral gene expression as in the case of Simian Vacuolating Virus 40 (SV40)-encoded miRNA (Sullivan et al., 2005). Mir-GpSGHV_170050R was identified in both asymptomatic and symptomatic *G. pallidipes* by the NGS approach. Although the direct viral target of this virus-encoded miRNA was a hypothetical GpSGHV protein (GpSGHV ORF132), the identification of a GpSGHV-encoded miRNA during asymptomatic infections may suggest a role in maintaining latent infection in *G. pallidipes.* Similar observations were made for *Heliothis zea* nudivirus-1 (HzNV-1), another large, rod-shaped, DNA insect virus that encodes miRNAs to promote latent infections by inhibiting viral gene expression (Wu et al., 2011).

Prediction of putative target transcripts of the differentially expressed host and GpSGHV-encoded miRNAs may help in understanding the transcriptional regulation of genes depending on whether the GpSGHV infection becomes symptomatic or remains asymptomatic. The targets of the 15 differentially expressed miRNAs were predicted from the 3′-UTR’s of the 6,071 available *G. pallidipes* transcripts in the VectorBase (Giraldo-Calderón et al., 2014). The potential target transcripts were classified into different categories according to GO annotations. Our study focused on the immune related genes targeted by the regulated host miRNAs and the virus-encoded miRNAs for the GO enrichment (i.e., biological process, molecular function or cellular component) and pathway analysis. A single miRNA might regulate multiple target genes and even regulate the same target gene at multiple sites (Skalsky and Cullen, 2010). In the current study one of the most up-regulated host miRNA (i.e., miR-184-3p) according to the NGS data was found to target most all of the immune genes predicted to be targeted by the complete set of up-regulated miRNAs. Most of these targeted immune genes appear to be involved in the Imd and Toll pathways, which are known to play a role in antiviral immunity in insects (Kingsolver et al., 2013), for example against arbovirus infections (Xi et al., 2008; Avadhanula et al., 2009). Some of the targeted immune genes include the *FBN1* (glycoprotein involved in cell communication), *Rab27* and *Apoltp* (involved in positive regulation of lipid transport) and these were indeed down-regulated in the symptomatic flies. In baculovirus-infected *S. frugiperda* cells, where miR-184-3p was up-regulated, target prediction and transcript level analysis showed that this miRNA may either positively or negatively regulate particular target gene transcripts (Mehrabadi et al., 2013). Notably, the *Apoltp* and *VtgR* were predicted to be involved in vitellogenesis, the main process in oogenesis and egg production. The down-regulation of these genes may explain the ovarian abnormalities and reduced reproductive fitness observed in symptomatically infected female tsetse flies. Similar observations have been reported in the housefly, *Musca domestica*, whereby MdSGHV infections which causes similar SGH syndrome in their host, were found to suppress vitellogenesis by blocking the transcription of hexamerin and yolk proteins and cause shut down of oogenesis and hence reduce reproduction (Kariithi et al., 2017).

Multiple miRNAs might co-regulate one target gene at the same time (Skalsky and Cullen, 2010). For instance, in our study the *CEP* gene, which was up-regulated in symptomatic *G. pallidipes*, contained target sites for two up-regulated miRNAs (miR-184-3p and miR-263-5p). This also indicates that although most reports show that miRNA-target interaction lead to negative regulation of the target gene (Asgari, 2011), a positive regulatory effect may also occur by promoting transcript stabilization or translation as previously reported (Hussain et al., 2012; Conrad et al., 2013). Approximately, 30 % of the immune genes targeted by the up-regulated host miRNAs were found to be also targets of the GpSGHV-encoded miRNAs, with most of these genes involved in Toll pathway signaling. The Toll pathway is known to direct antiviral defense in DENV-infected *Ae. albopictus* following down-regulation of the host miRNA mir-375 (Liu et al., 2015). The GpSGHV-encoded miRNAs targeted transcripts of *Rab27*, *FBN1* and the *Apoltp* genes, transcripts that can also be targeted by host miR-184-3p. In addition, these viral miRNAs also specifically targeted the thyroid receptor–interacting protein (*TRIPB*), serine protease 1 *(Ser1)* and midgut trypsin *(Mid-Try)*. Trypsin is involved in serine endopeptidase activity and has been reported to cleave the well conserved baculovirus P74, a viral attachment protein. This p74 cleavage is crucial for infection and necessary for the baculovirus to establish a primary infection in midgut cells (Slack et al., 2008). It should be stressed here that GpSGHV encodes a homolog of the baculovirus P74 protein (Abd-Alla et al., 2008). Why the virus up-regulates trypsin in already infected insects is not clear.

In this study, attempts to artificially up-regulate miR-184-3p by injecting its mimic led to increased expression levels of the GpSGHV *odv-e66* gene. However, the corresponding miR-184-3p inhibitor did not cause any significant difference in GpSGHV *odv-e66* expression. Since miR-184-3p may regulate transcripts of several host genes, how this affects GpSGHV infection requires further studies. Notably, miR-184-3p has been reported to be induced by Interleukin-22 (IL-22), an inflammatory cytokine, by down-regulating the expression of Argonaute-2 (AGO-2), a key protein of the RNAi pathway (Roberts et al., 2013). RNAi is an important immune defense pathway against virus infections in most insects (Van Rij, 2008); the up-regulation of miR-184-3p in symptomatically GpSGHV infected flies may responsible to modulate *AGO-2* expression and thereby regulate virus replication. This agrees with observations for invertebrate iridovirus (IIV-6) in *Drosophila* (Bronkhorst et al., 2012) and our previous study where we found *AGO-2* to be down-regulated in symptomatically infected flies compared to asymptomatically infected flies (Meki et al., in press).

## Conclusion

In the current study, we have identified host and viral-encoded miRNAs and evaluated their expression profiles during GpSGHV asymptomatic and symptomatic infections in *G. pallidipes*. Fifteen differentially expressed host miRNAs were identified and their target predictions suggested that miR-184-3p, the most up-regulated miRNA in symptomatic flies, might be involved in regulating immune responses and oogenesis and hence the reproductive fitness of the flies, since it targeted mostly immune related and vitellogenesis genes. This study further presents the first evidence that GpSGHV alters the host miRNA profile in *Glossina*, a finding that provides a baseline for further investigations to understand the GpSGHV-*Glossina* interactions. Finally, the data from the current study provides insights into the interaction between GpSGHV and *G. pallidipes* miRNAs, and provides potential avenues to further study the mechanisms of immune response during GpSGHV infections in tsetse fly. This information may provide strategies to control GpSGHV infections in tsetse mass rearing facilities, a prerequisite to SIT implementation, by utilization of particular miRNAs, especially those implicated in anti-viral responses, or by inhibition of pro-viral miRNAs by mir inhibitors. Alternatively, these miRNAs might be overexpressed in symbionts (*Sodalis glossinidius*) via paratransgenesis (De Vooght et al., 2014, 2012).

## Availability of Data and Materials

Materials described in the manuscript, including all relevant raw data, will be freely available to any scientist wishing to use them for non-commercial purposes upon request via e-mail with the corresponding author.

## Author Contributions

AA and II designed the research setup. AA, JV, HK, and MO, supervised the Ph.D. candidate IM for experimental parts involved this research. IM, AP, AA, DB, OO, and II conducted the experiments, collected and analyzed data and prepared the figures. IM, AP, HK, MO, AA, JV, DB, and II participated in the writing of the manuscript. All authors have read and agreed to the contents of the final manuscript and confirm that the manuscript conforms to the journal’s policies.

## Conflict of Interest Statement

The authors declare that the research was conducted in the absence of any commercial or financial relationships that could be construed as a potential conflict of interest.

## Footnotes

1 https://www.qiagenbioinformatics.com

2 https://www.vectorbase.org

3 http://rfam.xfam.org

4 http://rna.tbi.univie.ac.at/cgi-bin/RNAWebSuite/RNAfold.cgi

5 https://cm.jefferson.edu/rna22v2/

6 http://bibiserv.techfak.uni-bielefeld.de/rnahybrid

7 https://www.blast2go.com

8 http://bordensteinlab.vanderbilt.edu/IIID/test_immunity.php

9 http://www.cytoscape.org

## Supplementary Material

TABLE S1 Sequences for the primers used for expression analysis by RT-qPCR and the miR-184-3p mimic and inhibitor sequences.

TABLE S2 Sequencing reads matched to the GpSGHV-UGA genome. The location, length of the sRNA reads, as well as the ORF of their origin on the GpSGHV genome are shown. The green highlighted reads were identified as GpSGHV-encoded miRNAs based on the pre-miRNA prediction (i.e., the presence of mature miRNA and mfe <−20 kcal/mol) and the secondary structure analysis.

TABLE S3 Host miRNAs identified in sRNA libraries of asymptomatically and symptomatically infected *G. pallidipes*. The mature miRNA sequence, the miRNA gene families, total reads of each of the identified miRNA and their normalized expression levels are shown.

TABLE S4 Predicted transcripts modulated by up-, down-regulated and GpSGHV-encoded miRNAs. Gene targets of the **(A)** up- and **(B)** down-regulated host miRNAs, including the miRNA binding site/position on the transcript, the minimum free energy of the miRNA-target interaction and their secondary structures. **(C)** The immune related transcripts targeted by the up-regulated miRNAs with the immune pathways involved and the bit score as predicted on the Insect Innate Immunity Database. **(D)** Gene targets of the GpSGHV-encoded miRNAs. **(E)** The top 10 genes (lowest *P*-value and minimum mfe) targeted by the up- and down-regulated host miRNAs and the GpSGHV-encoded miRNAs.
