# Supplementary material for: Expression Profile of Glossina pallidipes MicroRNAs During Symptomatic and Asymptomatic Infection With Glossina pallidipes Salivary Gland Hypertrophy Virus (Hytrosavirus)
Source: Front Microbiol. 2018 Sep 3;9:2037. doi: 10.3389/fmicb.2018.02037 (PMC6129597; doi:10.3389/fmicb.2018.02037)
Supplement: TABLE S1 — Sequences for the primers used for expression analysis by RT-qPCR and the miR-184-3p mimic and inhibitor sequences. [file Table_1.DOCX]

**Supplementary Table 1:** Sequences for the primers used for expression analysis by RT-qPCR and the miR-184-3p mimic and inhibitor sequences.

| **Target** | **Primer name** | **Sequence** |  |
| --- | --- | --- | --- |
| **Primers for the differentially expressed host miRNAs** | | | |
| miR-184-3p | miR-184-3p_F | AACTGGACGGAGAACTGATAAGGGC |  |
| miR-277-3p | miR-277-3p_F | TTGTAAATGCACTATCTGGTACGAC |  |
| miR-7-3p | miR-7-3p_F | CAACAAAATCACTAGTCTTCCA |  |
| miR-8-3p | miR-8-3p_F | TAATACTGTCAGGTAAAGATGTC |  |
| miR-999-3p | miR-999-3p_F | TGTTAACTGTAAGACTGTGTCT |  |
| miR-1-3p | miR-1-3p_F | TGGAATGTAAAGAAGTATGGAGCGA |  |
| miR-263-5p | miR-263-5p_F | AATGGCACTGGAAGAATTCACGG |  |
| miR-276-5p | miR-276-5p_F | AAGAGCACGGTATGAAGTTCCTA |  |
| miR-283-5p | miR-283-5p_F | AAATATCAGCTGGTAATTCTG |  |
| miR-6497 | miR-6497_F | CGTAACTTCGGGATAAGGATTGGCTCTGAAG |  |
| miR-9-3p | miR-9-3p_F | TCATACAGCTAGATAACCAAAGA |  |
| **Primers for the selected immune genes targeted by miRNAs** | | | |
| GPAI025158 | GPAI025158-qPCR_F | GTATTCCTCACACTTCCTCCAAC |  |
|  | GPAI025158-qPCR_R | CCACCATAACTGAGAACAGAAGAA |  |
| GPAI030501 | GPAI030501-qPCR_F | CGATGCTATGGGTTTTCTGCT |  |
|  | GPAI030501-qPCR_R | TCGCATTTATTACCGCACAACA |  |
| GPAI014544 | GPAI014544-qPCR_F | GGATGCGAGAACGGGAAATG |  |
|  | GPAI014544-qPCR_R | CAAACACTCTTCCTGACAAAATGG |  |
| GPAI038987 | GPAI038987-qPCR_F | CGGATTGGTTTAGTTTCGGTTG |  |
|  | GPAI038987-qPCR_R | CCACTTCTTCTCTTTTCACTTTCTC |  |
| GPAI034557 | GPAI034557-qPCR_F | TAATCGCTGGTTGGGTAATGAG |  |
|  | GPAI034557-qPCR_R | GTTTGTATCTATTCGGTTCCTCCT |  |
| GPAI025990 | GPAI025990-qPCR_F | GCAATACTTCCCTGTCCATAAC |  |
|  | GPAI025990-qPCR_R | CTGTCGTCCAACCTTCACTT |  |
| GPAI001218 | GPAI001218-qPCR_F | ATGAGGTGGATGAAAGTGATAAAGG |  |
|  | GPAI001218-qPCR_R | CTTCCTCGGGTATGTCAATCAAG |  |
| GPAI015640 | GPAI015640-qPCR_F | GCATACCTTTTCTGTTGGTTGG |  |
|  | GPAI015640-qPCR_R | CGAGTTTTGGCTGATGTTTCTAC |  |
| GPAI042543 | GPAI042543-qPCR_F | CAAATCACGCATAGCCACAAG |  |
|  | GPAI042543-qPCR_R | AATGGGTTTAGTGGAGGGTTTC |  |
| GPAI007448 | GPAI007448-qPCR_F | TGCGACAAAAGCTAGATGTAATGGG |  |
|  | GPAI007448-qPCR_R | AAATCCTCAAACACAGCACCAACA |  |
| **Primers for the virus and reference gene quantification** | | | |
| GpSGHV (*odv-e66*) | GpSGHVqPCR-F | CAAATGATCCGTCGTGGTAGAA |  |
|  | GpSGHVqPCR-F | AAGCCGATTATGTCATGGAAGG |  |
| *β-Tubulin* (tsetse) | Tse-TubqPCR-F | GATGGTCAAGTGCGATCCT |  |
|  | Tse-TubqPCR-R | TGAGAACTCGCCTTCTTCC |  |
| **Mir-184 mimic and inhibitor sequences** | | | |
| miR-184-3p | Mimic | UGGACGGAGAACUGAUAAGGGC |  |
|  | Inhibitor | GCCCUUAUCAGUUCUCCGUCCA |  |
